# Synthetic arylquinuclidine derivatives exhibit antifungal activity against *Candida albicans*, *Candida tropicalis *and *Candida parapsilopsis*

**DOI:** 10.1186/1476-0711-10-3

**Published:** 2011-01-21

**Authors:** Kelly Ishida, Juliany Cola Fernandes Rodrigues, Simon Cammerer, Julio A Urbina, Ian Gilbert, Wanderley de Souza, Sonia Rozental

**Affiliations:** 1 Laboratório de Biologia Celular de Fungos, Instituto de Biofísica Carlos Chagas Filho, Universidade Federal do Rio de Janeiro, Avenida Carlos Chagas Filho 373, Bloco C, Sala C0-026, Cidade Universitária, 21.941-902, Rio de Janeiro/RJ, Brazil; 2 Laboratório de Ultraestrutura Celular Hertha Meyer, Instituto de Biofísica Carlos Chagas Filho, Universidade Federal do Rio de Janeiro, Avenida Carlos Chagas Filho 373, Bloco C, Cidade Universitária, 21.941-902, Rio de Janeiro/RJ, Brazil; 3 Welsh School of Pharmacy, Cardiff University, Redwood Building, King Edward VII Avenue, Cardiff, CF10 3XF, UK; 4 Laboratorio de Química Biológica, Instituto Venezolano de Investigaciones Científicas, Caracas 1020, Venezuela; 5 Instituto Nacional de Metrologia, Normalização e Qualidade Industrial-Inmetro, Avenida Nossa Senhora das Graças 50, 25250-020, Xerém, Duque de Caxias/RJ, Brazil

## Abstract

**Background:**

Sterol biosynthesis is an essential pathway for fungal survival, and is the biochemical target of many antifungal agents. The antifungal drugs most widely used to treated fungal infections are compounds that inhibit cytochrome P450-dependent C14α-demethylase (CYP51), but other enzymes of this pathway, such as squalene synthase (SQS) which catalyses the first committed step in sterol biosynthesis, could be viable targets. The aim of this study was to evaluate the antifungal activity of SQS inhibitors on *Candida albicans*, *Candida tropicalis *and *Candida parapsilopsis *strains.

**Methods:**

Ten arylquinuclidines that act as SQS inhibitors were tested as antiproliferative agents against three ATCC strains and 54 clinical isolates of *Candida albicans*, *Candida tropicalis *and *Candida parapsilopsis*. Also, the morphological alterations induced in the yeasts by the experimental compounds were evaluated by fluorescence and transmission electron microscopy.

**Results:**

The most potent arylquinuclidine derivative (3-[1'-{4'-(benzyloxy)-phenyl}]-quinuclidine-2-ene) (WSP1267) had a MIC_50 _of 2 μg/ml for all species tested and MIC_90 _varying from 4 μg/ml to 8 μg/ml. Ultrathin sections of *C. albicans *treated with 1 μg/ml of WSP1267 showed several ultrastructural alterations, including (a) loss of cell wall integrity, (b) detachment of the plasma membrane from the fungal cell wall, (c) accumulation of small vesicles in the periplasmic region, (d) presence of large electron-dense vacuoles and (e) significantly increased cell size and cell wall thickness. In addition, fluorescence microscopy of cells labelled with Nile Red showed an accumulation of lipid droplets in the cytoplasm of treated yeasts. Nuclear staining with DAPI revealed the appearance of uncommon yeast buds without a nucleus or with two nuclei.

**Conclusion:**

Taken together, our data demonstrate that arylquinuclidine derivatives could be useful as lead compounds for the rational synthesis of new antifungal drugs.

## Background

Candidiasis is the most common fungal infection; it is found all over the world. *Candida *spp. isolates are the fourth most common microorganisms found in bloodstream infections and are particularly prevalent among patients hospitalised for long periods who have been exposed to antibiotics, immunosuppressive therapy, parenteral nutrition, and multiple invasive medical procedures [1]. Systemic fungal infections are generally difficult to diagnose and hard to treat, having an attributable mortality rate of near 40% [1]. Although *Candida albicans *is the species most frequently isolated from those patients, being responsible for more than half of candidiasis cases, the incidence of other species, such as *Candida parapsilopsis *and *Candida tropicalis*, is increasing [2].

Most current therapies to treat fungal infections are based on disrupting fungal membrane homeostasis. The most commonly used groups of antifungal agents are the polyenes (e.g., amphotericin B), which disrupt membrane function by direct association with fungal sterols, and the azoles (fluconazole, itraconazole, voriconazole and posaconazole), which inhibit sterol biosynthesis in a step catalysed by the cytochrome P450-dependent C14α-demethylase [3]. Treatment of invasive *Candida *infections is often complicated by high toxicity, low tolerability or a narrow spectrum of activity of the current antifungal drugs as well as an increase in the incidence of azole-resistant strains [4]. These difficulties have driven the search for new treatments with different mode(s) of action.

Squalene synthase (SQS) is a key enzyme in sterol biosynthesis that catalyses an unusual head-to-head condensation of two molecules of farnesyl pyrophosphate in a two-step reaction to produce squalene, which is the first committed step in sterol biosynthesis. Importantly, inhibition of this enzyme does not affect the biosynthesis of other essential isoprenoids derived from farnesyl pyrophosphate derivatives, such as ubiquinones, dolichols, haeme, and C_15_- or C_20_-isoprenoid chains [5]. In recent years, significant effort has been devoted to evaluating SQS with the aim of developing new cholesterol-lowering agents in mammalian cells [6]. Several classes of compound have been developed, including arylquinuclidines [7] and zaragozic acids. In addition, arylquinuclidine compounds have potent activity in concentrations varying from the nanomolar to subnanomolar range against parasitic protozoa, such as *Trypanosoma cruzi*, *Leishmania *and *Toxoplasma gondii *[8-12]. Zaragozic acids show significant activity against various fungal species [13].

In this work, the antifungal effect of the prototypical arylquinuclidine molecule BPQ-OH {3-(biphenyl-4-yl)-3-hydroxyquinuclidine or 3-biphenyl-4-yl-1-aza-bicyclo[2,2,2]-octan-3-ol} and 9 derivatives was evaluated against three ATCC strains and 54 clinical isolates of *C. albicans*, *C. parapsilosis *and *C. tropicalis*. Five aspects of the antifungal effects of these compounds were investigated: (i) growth inhibition, (ii) fungicidal effect, (iii) morphological alterations, (iv) lipid accumulation and (v) cell cycle alteration.

## Methods

### Drugs

**Figure 1:**
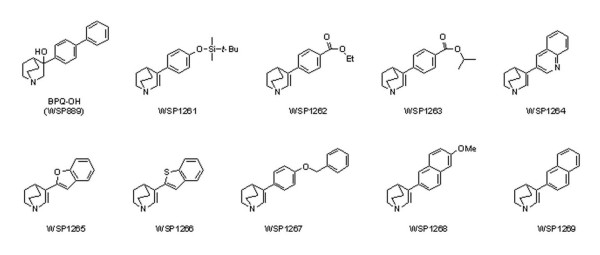
**Molecular structures of the arylquinuclidine derivatives used in this work. The arylquinuclidine ring is indicated by an arrow**.

The prototypical arylquinuclidine BPQ-OH was prepared as described by Brown et al. [14]. Nine analogues (WSP1261, WSP1262, WSP1263, WSP1264, WSP1265, WSP1266, WSP1267, WSP1268 and WSP1269) (Figure 1) that are active against *Leishmania major *and selective to the parasite's SQS over the human enzyme [15] were also tested. Fluconazole, itraconazole, and amphotericin B were used as standard antifungal drugs. The compounds were diluted in DMSO, except fluconazole, which was diluted in water. They were then maintained at -20°C.

### Yeast collection

To evaluate the antifungal activity of the synthetic arylquinuclidine derivatives, three standard strains from the American Type Culture Collection (ATCC) were used for screening: *Candida albicans *ATCC 10231, *Candida parapsilosis *ATCC 22019 and *Candida tropicalis *ATCC 13803. In addition, 54 clinical isolates of *Candida *were evaluated for high antifungal activity compounds: *Candida albicans *(n = 21), *Candida parapsilosis *(n = 19), and *Candida tropicalis *(n = 14). These strains were isolated between 2002 and 2006 at the Microbiology/Mycology Laboratory of Hemorio, Rio de Janeiro, Brazil, and kindly provided by Marcos Dornelas Ribeiro. The yeasts were maintained on Sabouraud dextrose agar at 4°C, and subcultures were performed prior to each experiment in the same medium for 48 h at 35°C.

### Antifungal activity assays

Minimum inhibitory concentration (MIC) assays were performed using a broth microdilution method adapted from the M27-A3/CLSI document [16]. Briefly, each compound was diluted in RPMI 1640 medium (Sigma Chemical Co., Missouri, USA), buffered with MOPS 0.16 M, pH 7.0, diluted in a 96-well microtitre tray and added to the growth medium to obtain the following final concentrations: 0.03-16 μg/ml (arylquinuclidines and itraconazole), 0.25-128 μg/ml (fluconazole) and 0.007-4 μg/ml (amphotericin B). Yeasts were then added to each well at the final concentration of 0.5-2.5 × 10^3 ^cfu/ml. The microtitre trays were incubated at 35°C for 48 h in a dark, humid chamber. Afterwards, IC_50 _values (the lowest concentrations that inhibit 50% of the yeast growth in comparison to control) for each compound were determined using a spectrophotometer at 492 nm. To determine the trailing effect in *Candida *strains (persistent growth of some isolates of *Candida *in drug concentrations above the IC_50 _after 48 h of incubation, commonly observed in broth microdilution tests with azole agents) visual reading was performed after 24 and 48 h of incubation. MIC_50 _and MIC_90 _values (minimum inhibitory concentration required to inhibit the growth of 50% and 90% of the population of each *Candida *species with n ≥ 10, respectively) were also determined for standard drugs. Only arylquinuclidine derivatives that showed antifungal activity lower than 16 μg/mL for ATCC strains were evaluated against the 54 clinical isolates. This breakpoint was selected because is the recommended concentration for the evaluation of new azoles without previously defined breakpoints [16].

Minimum fungicidal concentrations (MFC) were determined by transferring an aliquot (10 μl) of each sample treated with concentrations higher than the IC_50 _onto a drug-free Sabouraud dextrose agar plate and incubated at 35°C for 48 h. MFC means the lowest concentration of compound that produces no fungal growth. A fungicidal effect was considered when the MFC value was equal to or up to four times the IC_50 _value. Above this value, the antifungal effect was considered fungistatic [17].

### Fluorescence microscopy

*C. albicans *isolate 77 was chosen for all morphological analyses because this strain was susceptible to all standard antifungal drugs and had the median IC_50 _value for WSP1267 (1 μg/ml) of all the fungal populations tested. *C. albicans *isolate 77 untreated and treated with the IC_50 _of WSP1267 for 48 h at 35°C were washed in PBS, pH 7.2, fixed with 4% paraformaldehyde in PBS for 30 min, adhered to glass coverslips, covered with poly-L-lysine and incubated with different fluorophores: (a) 5 μg/ml Nile Red (Fluka, USA), a fluorescent lipophilic stain, for 30 min; or (b) 1 μg/ml DAPI (Sigma Chemical Co., Missouri, USA), a fluorescent stain that binds strongly to DNA, for 10 min. The coverslips were then mounted in n-propyl gallate solution and observed under a Zeiss Axioplan epifluorescence microscope equipped with rhodamine (Nile Red fluorescence) and DAPI filters. Images were recorded on a C5810 Hamamatsu camera. The number of altered *Candida *was determined after counting at least 300 yeasts. To analyse the effects on the cell cycle, three morphological phases were considered: (I) cells with one nucleus, (II) cells with one bud (daughter cell) and one nucleus in the mother cell, and (III) cells with one bud and two nuclei (one in each cell).

### Transmission electron microscopy

*C. albicans *isolate 77 untreated and treated with the IC_50 _of WSP1267 for 48 h at 35°C were fixed in a solution containing 2.5% glutaraldehyde and 4% freshly prepared formaldehyde in 0.1 M cacodylate buffer (pH 7.2) for 1 h. Then yeasts were post-fixed in 0.1 M cacodylate buffer (pH 7.2) containing 1% osmium tetroxide, 1.25% potassium ferrocyanide and 5 mM CaCl_2 _for 2 hours. After that, yeasts were serially dehydrated in ethanol and embedded in Spurr epoxy resin. Ultrathin sections were obtained with a Leica ultramicrotome, stained with uranyl acetate and lead citrate, and observed under a Zeiss CEM-900 electron microscope.

### Cytotoxicity assays

Green monkey kidney (Vero) cells were used for the cytotoxicity assays. Cells were maintained in Dulbecco's Modified Eagle's Medium (DMEM, Gibco Invitrogen Corporation, New York, USA) supplemented with 2 mM L-glutamine, 10% heat-inactivated foetal bovine serum (FBS), and 50 μg/ml gentamicin at 37°C in a 5% CO_2_. For the experiments, Vero cells (2.5 × 10^4 ^cells/well) were dispensed into a 96-well microtitre tray and incubated for 24 h to obtain a monolayer. Monolayers of Vero cells were treated with concentrations of WSP1267 varying from 1 μg/ml to 40 μg/ml for 48 h at 37°C in 5% CO_2_. The monolayers were fixed in 10% trichloroacetic acid for 1 h at 4°C and stained with sulphorhodamine B for 30 min at 4°C. The optical densities were obtained in a spectrophotometer at 530 nm to calculate the 50% cytotoxic concentration (CC_50_) [18].

### Statistical analysis

Statistical analyses were performed with GraphPad Prism 5.0 (GraphPad Software), and p < 0.05 was considered significant. Student's t-test was used to analyse the morphological differences between untreated and treated yeasts. The frequency distribution of IC_50 _values was calculated to determine differences between the MIC_50 _and MIC_90 _values

## Results

### Antifungal activity

Among all quinuclidines tested, WSP1267 (3-[1'-{4'-(benzyloxy)-phenyl}]-quinuclidine-2-ene) exhibited the best anti-*Candida *effect, with IC_50 _varying from 0.5 μg/ml to 8 μg/ml (*C. tropicalis *ATCC 13803: 0.5 μg/ml; *C. albicans *ATCC 10231: 1 μg/ml; *C. parapsilosis *ATCC 22019: 8 μg/ml). All other quinuclidine analogues tested on these three ATCC strains had IC_50 _values higher than 16 μg/ml.

Afterwards, the anti-*Candida *effect of WSP1267 was evaluated against 54 *Candida *spp. clinical isolates (21 *Candida albicans*, 19 *Candida parapsilosis *and 14 *Candida tropicalis*). Although the IC_50 _values varied from 0.5 μg/ml to 16 μg/ml (Table 1), the MIC_50 _values (median value of each population) were the same for all tested *Candida *species (2 μg/ml). MIC_90 _values were 4 μg/ml for *C. parapsilosis*, 8 μg/ml for *C. albicans *and *C. tropicalis *(Table 1). For this collection of strains, *C. parapsilosis *clinical isolates were more susceptible to WSP1267 and to standard antifungal agents, especially to itraconazole and amphotericin B, than the other two species.

**Table 1:** Minimum inhibitory concentration of WSP1267, fluconazole, itraconazole and amphotericin B against 54 *Candida *spp. clinical isolates.

Drugs	All species (n = 54)	*C. albicans* (n = 21)	*C. parapsilosis* (n = 19)	*C. tropicalis* (n = 14)
**WSP1267**				
Range	0.5-16	0.5-8	0.5-8	2-16
Geometric mean	2.54	2.2	2.46	3.32
MIC_50_	2	2	2	2
MIC_90_	8	8	4	8
**Fluconazole**				
Range	<0.25 to >128^a^	<0.25 to 2	<0.25 to 1	<0.25 to >128^a^
Geometric mean	0.58	0.48	0.61	0.65
MIC_50_	0.25	0.25	0.5	0.5
MIC_90_	1	1	1	4
**Itraconazole**				
Range	<0.03 to >16^b^	<0.03 to 0.5	<0.03 to 0.5	<0.03 to >16^b^
Geometric mean	0.05	0.09	0.05	0.05
MIC_50_	0.03	0.03	0.03	0.03
MIC_90_	0.06	0.12	0.03	0.03
**Amphotericin B**				
Range	0.007-0.5	0.007-0.25	0.007-0.25	0.007-0.5
Geometric mean	0.04	0.05	0.03	0.04
MIC_50_	0.03	0.03	0.03	0.06
MIC_90_	0.12	0.12	0.25	0.12

(MIC values are expressed in μg/ml)

^a ^Resistant (two *C. tropicalis*) and trailing effect (nine *C. albicans*, four *C. tropicalis*, and three *C. parapsilosis*)

^b ^Resistant (three *C. tropicalis*, one *C. albicans*), susceptibility dependence-dose (three *C. albicans*, two *C. tropicalis *and one *C. parapsilosis*) and trailing effect (six *C. albicans*, four *C. tropicalis *and one *C. parapsilosis*).

The MFC values for WSP1267 were higher than 16 μg/ml for all clinical isolates (data not shown), discounting any possibility of a fungicidal aspect of this drug.

All the clinical isolates showed similar susceptibility profile to amphotericin B (IC_50 _range of 0.007-1 μg/ml). Two isolates of *C. tropicalis *were resistant to fluconazole, while 16 exhibited trailing effects (9 *C. albicans*, 4 *C. tropicalis*, and 3 *C. parapsilosis*). In addition, 4 isolates were resistant to itraconazole (3 *C. tropicalis *and 1 *C. albicans*), 6 isolates showed susceptibility in a dose-dependent manner (3 *C. albicans*, 2 *C. tropicalis *and 1 *C. parapsilosis*), and 11 isolates presented trailing effects (6 *C. albicans*, 4 *C. tropicalis *and 1 *C. parapsilosis*).

Remarkably, all isolates resistant to fluconazole (IC_50 _>64 μg/ml) and itraconazole (IC_50 _>16 μg/ml) were susceptible to WSP1267 with a mean IC_50 _value of 8 μg/ml.

### Morphological and ultrastructural effects

**Figure 2:**
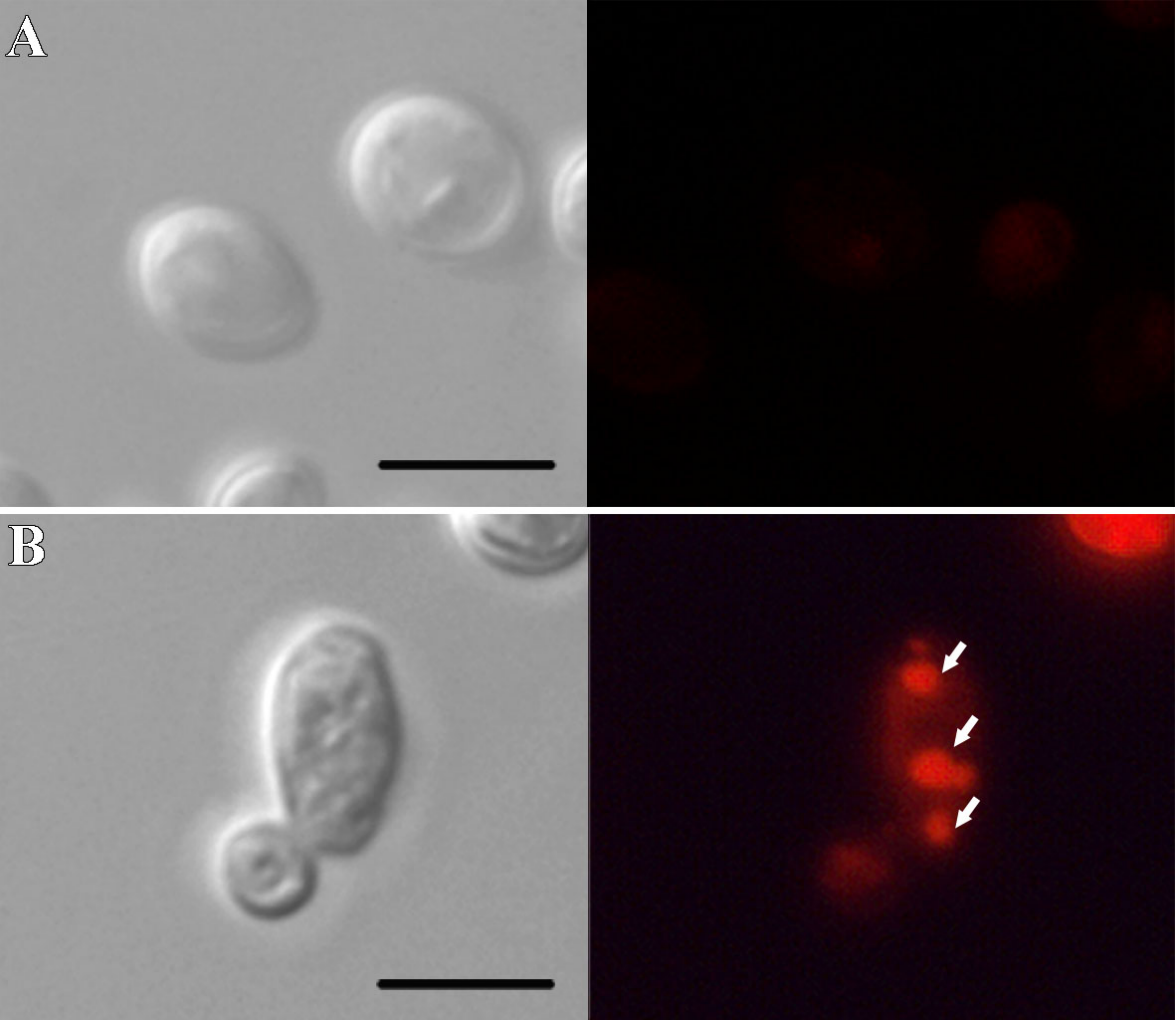
**Differential interference contrast (DIC) microscopy (left) and fluorescence microscopy with Nile Red (right) of *C. albicans *(isolate 77) untreated (A) and treated with 1 μg/ml WSP1267 [IC**_**50**_**] for 48 h at 35°C (B)**. The treatment induced an accumulation of lipid droplets in the cytoplasm of the yeasts (white arrows in B), which is not present in the untreated yeasts. Bars = 5 μm.

Treatment of *C. albicans *with WSP1267 caused morphological and ultrastructural effects. Fluorescence microscopy of WSP1267-treated yeasts incubated with Nile Red revealed an accumulation of lipid droplets in the cytoplasm of treated yeasts when compared to *C. albicans *(isolate 77) control (Figure 2A-B), indicating that WSP1267 can induce the accumulation of lipids and/or precursors.

**Figure 3:**
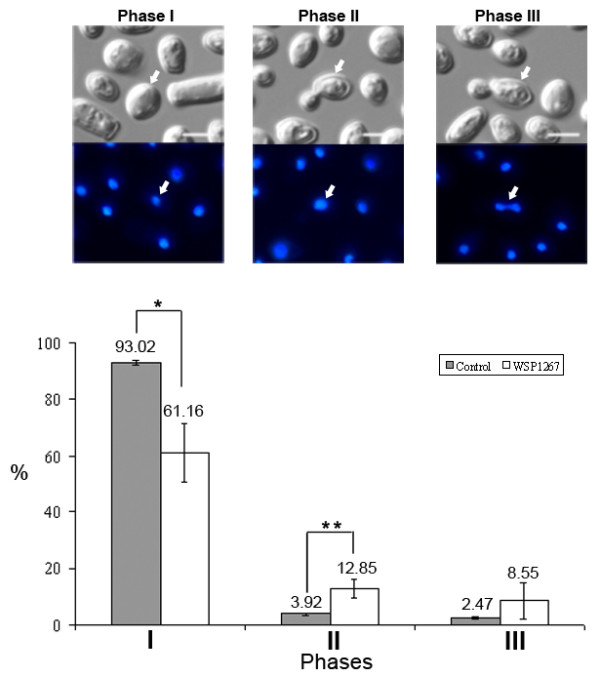
**DIC microscopy (above) and fluorescence microscopy with DAPI (below) of *C. albicans *(isolate 77) untreated in different phases of cell cycle**. Treatment with 1 μg/ml WSP1267 [IC_50_] for 48 h at 35°C induced alterations in the cell cycle (white bars) in comparison with untreated yeasts (grey bars). The graphic revealed a significant increase in the number of yeasts with alterations in the cell cycle (decrease in phase I and increase in phases II and III). Cell cycle phases: (I) cells with one nucleus, (II) cells with bud and one nucleus in the mother cell, and (III) cells with bud and two nuclei (one in each cell). Relevant yeast from each phase is indicated by white arrows. Bars = 5 μm; * p < 0.05; ** p < 0.01.

**Figure 4:**
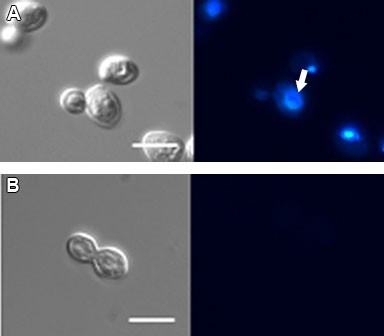
**DIC microscopy (left) and fluorescence microscopy with DAPI (right) of *C. albicans *(isolate 77) treated with 1 μg/ml of WSP1267 [IC**_**50**_**] for 48 h at 35ºC, showing abnormal chromatin condensation (A, white arrow) and absence of a nucleus (B). Bars = 5 μm**.

Treatment with the IC_50 _of WSP1267 induced alterations in the nuclear profile of *C. albicans *(Figure 3). The treatment induced a decrease of 34% (p < 0.05) in the number of yeasts presenting only one nucleus and no buds (phase I), an increase of three times (p < 0.01) in the number of yeasts with buds lacking nuclei and only the mother cell having a nucleus (phase II) and an increase of three times in yeasts with one bud and two nuclei (one in each cell) (phase III), but not statistically significant. Labelling with DAPI also revealed abnormal chromatin condensation in approximately 11% of yeasts (Figure 4A, white arrow), while no nucleus was observed in 9% of yeasts (Figure 4B).

**Figure 5:**
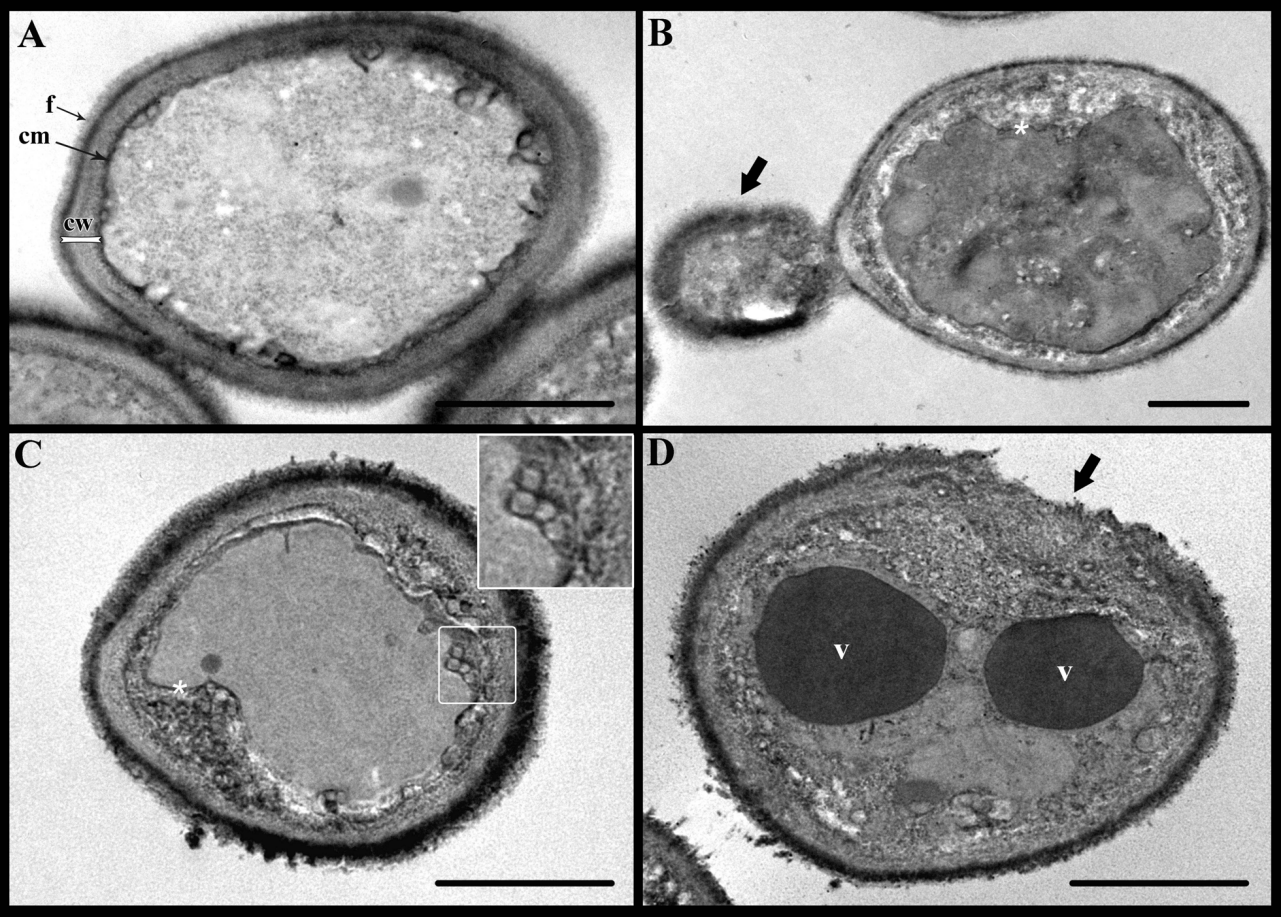
**Ultrathin sections of *C. albicans *(isolate 77) control and treated with 1 μg/ml of WSP1267 [IC_50_] for 48 h at 35°C.** Untreated yeast (A) present a preserved ultrastructure (fibrillar structures - f, cytoplasm membrane - cm, and compact cell wall - cw). However, in treated yeasts (B-D), different alterations can be observed: disruption of cell wall and loss of cell wall integrity in the bud (B, black arrow), detachment of cytoplasmic membrane from the cell wall (B-C, arrow), presence of small vesicles in the periplasmic region (3B-D and inset in 3C) and large electron-dense vacuoles [v] (Figure 3D). Bars = 1 μm.

The morphological effects of the treatment with the IC_50 _of WSP1267 were also evaluated using transmission electron microscopy, which revealed that some organelles and structures could be potential targets for this arylquinuclidine derivative. Ultrathin sections of untreated *C. albicans *showed well-preserved cells with an external fibrillar layer (f), a compact cell wall (cw), a plasma membrane with normal shape (cm) and a homogeneous cytoplasm (Figure 5A). After the treatment, different ultrastructural alterations were observed, including (i) detachment of the plasma membrane from the cell wall (Figures 5B-C, asterisks), (ii) the presence of small vesicles in the periplasmic region (Figure 5C, higher magnification in the inset), (iii) accumulation of large electron-dense vacuoles in the cytoplasm (Figure 5D, v), and (iiii) disruption of the cell wall (black arrow in Figure 5D). In addition, the loss of cell wall integrity was also observed in the bud (Figure 5B, black arrow). WSP1267 also induced a significant increase of cell size of *C. albicans *from 4.9 ± 0.5 μm (control cell) to 5.9 ± 0.08 μm (treated cell) (p < 0.01) and cell wall thickness from 234 ± 26 nm (control cell) to 303 ± 87 nm (treated cell) (p < 0.01).

### Cytotoxicity assays

A CC_50 _of 12 μg/ml was observed in Vero cells treated with WSP1267.

## Discussion

The incidence of fungal infections by *Candida *spp. in Brazilian medical centres is close to 2.5 cases per 1,000 admissions; the most prevalent species is *C. albicans*, followed by *C. tropicalis *and *C. parapsilosis *[1]. In addition, there is an important increase in the number of *Candida *clinical isolates that are resistant to azoles, the most important class of drugs to treat fungal infections [1,19-21]. In our study, the percentage of fluconazole-resistant isolates (3.7%) was lower than that observed in other studies on Brazilian clinical isolates (5.7%) [1], isolates from North America (10.2%) [19], and isolates from Europe (5.2%) [20]. Pinto et al. [21] used 37 strains isolated from different anatomical sites of 11 immunocompromised hospitalised patients infected with HIV from the Hospital of the Federal University of Espírito Santo (ES, Brazil) and reported four *Candida *strains resistant to amphotericin B (10.8%) and two to itraconazole (5.4%). In our work, a higher incidence of itraconazole-resistant isolates (7.4%) was observed, whereas all isolates were susceptible to amphotericin B, as shown in previous studies [1,19,20].

Examples of recent approaches to antifungal treatments include new triazoles such as ravuconazole and albaconazole, which display a broad-spectrum activity against yeast and filamentous fungi, including species resistant to fluconazole and itraconazole [22]. In addition, the use of inhibitors from other steps of sterol biosynthesis is an alternative approach to the development of new chemotherapeutic agents. Several inhibitors of the mevalonate and isoprenoid pathways, as well inhibitors of the steps exclusively involved in ergosterol biosynthesis, have been studied against fungi [23] and parasitic protozoa [24]. For example, fluvastatin, a synthetic 3-hydroxy-3-methylglutaryl-coenzyme A reductase inhibitor, developed as a cholesterol-lowering agent, has a synergistic and fungicidal effect in combination with fluconazole and itraconazole against *C. albicans*, *C. tropicalis*, *C. parapsilosis*, and *Cryptococcus neoformans*, including fluconazole-resistant strains [25]. Inhibitors of sterol 24-methyltransferase, an enzyme that catalyses the incorporation of a methyl group at position 24 in the sterol side chain in fungi and trypanosomatids, has a potent effect against different clinical isolates of *Candida *spp. [26].

In the present work, we decided to investigate the effects of arylquinuclidine-based SQS inhibitors as a potential strategy for candidiasis treatment based on previous studies conducted with trypanosomatid parasites [24]. BPQ-OH was one of the first arylquinuclidines (originally developed as a potential cholesterol-lowering agent) reported to have potent activity against the *Leishmania major *SQS [27], blocking sterol biosynthesis and the growth of *Leishmania *spp. and *T. cruzi *in the low-nanomolar to subnanomolar range [9-12,28]. However, BPQ-OH is not active against *Candida *spp. ATCC and clinical isolates. Therefore, we decided to test other derivatives that possessed a high activity and selectivity to the *L. major *enzyme over the human enzyme [15]. From the collection of arylquinuclidine compounds investigated in this study, only WSP1267 showed activity against *Candida *spp. with MIC_50 _and MIC_90 _values of 2 and 4-8 μg/ml, respectively. WSP1267 has also shown good potency against the *Leishmania major *SQS (IC_50 _= 0.1 μM) and activity against *L. donovani *parasites in cell culture (EC_50 _= 1.1 μM) [15]. Cytotoxicity studies carried out with mammalian cells by Cammerer et al. [15] revealed that WSP1267 presents a 50% cytotoxic concentration of 50% (CC_50_) of 13.7 μg/ml (47.2 μM) to L-6 cells. Our data using Vero cells to determine the cytotoxicity showed a similar value (12.1 μg/ml or 41.6 μM).

To analyse morphological alterations, two different approaches were used in this work: (i) labelling of lipid droplets and DNA with specific fluorescent dyes (Nile red and DAPI, respectively); and (ii) visualisation of general ultrastructural alterations by transmission electron microscopy. The *C. albicans *77 strain was chosen for all morphological analyses as a representative strain because it was susceptible to all standard antifungal drugs and presented the average WSP1267 IC_50 _value of all the fungal populations tested.

The presence of lipid droplets in the treated yeasts visualised by fluorescence microscopy using Nile Red (Figure 2) was correlated with the presence of electron-dense vacuoles observed by electron microscopy (Figure 5), indicating that the quinuclidine treatment may induce an accumulation of lipid precursors in the cytoplasm which could be interfering directly with the fungal growth. These lipid droplets have also been observed after treatment of *L. amazonensis *with quinuclidine derivatives, due a total depletion of endogenous sterols and concomitant accumulation of exogenous cholesterol, which is not sufficient to maintain the viability of the parasites [10]. Recently, our group has also shown a lipid accumulation in *C. albicans *clinical isolates after treatment with inhibitors of 24-sterol methyltransferase, another enzyme of the sterol biosynthesis pathway [26]. Moreover, previous studies show that it is also possible that quinuclidines interfere with fatty acid and phospholipid biosynthesis, as observed previously with mammalian cells treated with SQS inhibitors [29] and with the trypanosomatid *Chritidia deanei *after treatment with sterol 24-methyltransferase inhibitors [30].

Analyses of the effect of WSP1267 treatment on the *C. albicans *77 isolate cell cycle revealed that the number of yeasts containing one nucleus (phase I) was significantly reduced, with a concomitant increase in the number of cells containing one bud and one nucleus (phase II) and yeasts containing one bud and two nuclei (phase III), in comparison with non-treated yeasts. There might be two possible explanations for these phenotypes: (i) the cells do not complete cell division due to alterations in the lipid composition, which is essential for membrane structure and also for control of the cell cycle; or (ii) the cells do not finish nuclear division, producing buds without nuclei. Taken together with the low MIC values and high MFC values, these data suggest that the action of WSP1267 was mostly fungistatic. Quinuclidine derivatives and 24-sterol methyltransferase inhibitors have similar effects on the cell cycle of *L. amazonensis *promastigotes [10] and *Candida *spp. [26]. Moreover, previous work has demonstrated that sterols control certain kinases involved in the cell cycle of yeasts [31]. Some images in this study suggested that the treated yeasts presented abnormal chromatin condensation, which is characteristic of apoptotic cell death. This is consistent with a previous study demonstrating that the deletion of the SQS gene in *Saccharomyces cerevisiae *leads to apoptotic cell death due to an impairment of ergosterol biosynthesis [32].

Transmission electron microscopy revealed important alterations in the integrity of the cell wall and plasma membrane, which could be related to depletion of essential endogenous sterols necessary to maintain their structures. The presence of small vesicles in the periplasmic region was also observed, as was the accumulation of large electron-dense vacuoles, which are consistent with the images taken by fluorescence microscopy. Similar morphological alterations have been reported in *L. amazonensis *treated with SQS inhibitors: an increase in the number of lipid inclusions displaying different shapes and electron densities, which were associated with an intense disorganisation of the cellular membrane [10,28]. Moreover, the important ultrastructural alterations observed in *C. albicans *treated with WSP1267 have been seen in *C. albicans *treated with 24-sterol methyltransferase inhibitors [26] and are probably related to the impairment of ergosterol biosynthesis, as has been observed after treatment of *L. amazonensis *with other quinuclidine derivatives [10,28]. Additionally, previous work has shown that *Candida *spp. treated with azole agents present similar ultrastructural alterations under transmission electron microscopy [33-36]. Although only one *C. albicans *clinical isolate (77) was chosen to illustrate the morphological aspects caused by the use arylquinuclidine derivatives, others *C. albicans *and non-*albicans *strains, including those resistant strains to azoles, were also evaluated and similar morphological alterations were observed (data not shown).

## Conclusion

Our results show that the arylquinuclidine derivative WSP1267 effectively inhibited the growth of a collection of *Candida *clinical isolates, including several azole-resistant strains, probably due to impaired sterol biosynthesis, which led to inhibition on cell growth and the accumulation of lipid precursors in the cytoplasm. Although the antifungal activity of WSP1267 seems to be due to SQS inhibition, further studies are needed to identify the molecular target of WSP1267 in yeasts. Additionally, we observed effects on the integrity of the cell wall and plasma membrane and effects on the cell cycle, which are essential for cell viability. These data warrant further investigation of arylquinuclidines as lead compounds for the rational synthesis of new, more effective antifungal agents with fewer toxic effects.

## Competing interests

The authors declare that they have no competing interests.

## Authors' contributions

KI carried out all experiments and data analysis. KI, JCFR, JAU and SR participated in study design and manuscript writing. SC and IG collaborated in the synthesis of arylquinuclidine compounds. All authors have read and approved the final manuscript.
